# Sealing Efficacy of Single-cone Obturation Technique with MTA and CEM Cement: An in Vitro Bacterial Leakage Study

**DOI:** 10.5681/joddd.2014.014

**Published:** 2014-06-11

**Authors:** Mohammad Samiei, Mohammad Aghazade, Farrokh Farhadi, Naeimeh Shahveghar, Ali Torab, Seyyed Mahdi Vahid Pakdel

**Affiliations:** ^1^Assistant Professor, Department of Endodontics, Faculty of Dentistry, Tabriz University of Medical Sciences, Tabriz, Iran; ^2^Assistant Professor, Department of Microbiology, Faculty of Medicine, Tabriz University of Medical Sciences, Tabriz, Iran; ^3^Assistant Professor, Department of Oral and Maxillofacial Surgery, Faculty of Dentistry, Tabriz University of Medical Sciences, Tabriz, Iran; ^4^Under-graduate Student, Student Research Committee, Faculty of Dentistry, Tabriz University of Medical Sciences, Tabriz, Iran; ^5^Post-graduate Student, Department of Prosthodontics, Faculty of Dentistry, Tabriz University of Medical Sciences, Tabriz, Iran

**Keywords:** CEM cement, mineral trioxide aggregate, root canal therapy, root canal filling materials, single-cone obturation

## Abstract

***Background and aims.*** Various materials and methods have been introduced for obturating cleaned and shaped root canal systems. This in vitro study aimed to evaluate the sealing ability of single-cone obturation technique with mineral trioxide aggregate and calcium-enriched mixture based on bacterial leakage approach.

***Materials and methods.*** Sixty-four single-canal teeth were prepared and randomly divided into 5 groups, consisting of three experimental groups (n = 16) and two control groups (n = 8).In group 1, root canal obturation was performed using gutta-percha with 0.02 taper and AH26 sealer by lateral compaction technique. In groups 2 and 3, single Protaper gutta-percha cone was used for obturation with MTA and CEM cement, respectively. A bacterial leakage apparatus was utilized for leakage assessment for 60 days. Leakage comparison between the experimental groups was performed by one-way ANOVA using SPSS 16 statistical software.

***Results.*** The mean bacterial leakage intervals in groups 1, 2 and 3 were 33.68 ± 9.39, 40.68 ± 11.03 and 39.56 ± 9.03 days, respectively. One-way ANOVA indicated no significant differences in bacterial leakage between the three experimen-tal groups (P = 0.109).

***Conclusion.*** Single-cone obturation with well-fitted gutta-percha and MTA and CEM cement is an appropriate alternative for traditional lateral compaction technique.

## Introduction

Obturation of the cleaned and shaped root canal system is a critical step in root canal therapy to inhibit the penetration of bacteria and their by-products into the cleaned and disinfected root canal system, as well as preventing the recolonization of bacteria remaining after root canal therapy. Providing a filling in the root canal capable of sealing the coronal, apical, and lateral openings is one of the main treatment objectives. Sealing the root canal system relies on the adequate adaptation of a filling material to obliterate the canal space and its intricacies: fins, deltas, isthmuses and lateral canals.^1-3^ Obturation of the root canal system hermetically, both apically and coronally, prevents leakage and contamination of the root canal space.^4,5^

Lateral compaction of gutta-percha cones remains widely accepted as a benchmark compared to other root filling techniques, as it is a simple and reliable technique that can be applied to most cases.^6^ However, it may leave gaps between gutta-percha cones, sealer and canal walls and there is a risk of vertical root fractures during compaction.^7^

Filling of root canals optimally in three dimensions after cleaning and shaping is paramount in preventing re-infection of the root canal space.^8^ Single-cone techniques performed with conventional sealers have been perceived to be less effective in sealing root canals than the gutta-percha warm vertical compaction technique.^9^ However, non-compaction, single-cone filling of root canals has recently been revived with the introduction of master cones with greater taper that match the geometry of nickel–titanium instrumentation systems.^10^ The advent of contemporary root canal sealing systems, claimed to create bonds along the sealer–gutta-percha interface by modifications of the sealer or the root-filling material, may also support the use of a single-cone obturation technique. ^11^Limited information, however, is available on the sealing quality of these new single-cone root fillings as compared with that of warm vertical compaction of gutta-percha. Recently however, gutta-percha cones with increased taper have been developed because use of gutta-percha cones with the same taper as that of nickel-titanium instruments may reduce microleakage.

Mineral trioxide aggregate has superior sealing ability and biocompatibility in comparison to other root-end filling materials.^12^ The results of microleakage studies revealed that MTA assures the best apical seal.^13,14^ Asgary et al introduced a novel endodontic cement with clinical applications similar to those of MTA.^15^ It has been shown that this material provides acceptable seal, similar to MTA and superior than IRM.^16^ The cement is biocompatible, induces hard tissue formation, forms an effective seal against entrance of microorganisms, is able to set in an aqueous environment, has antibacterial effects is resistant to washout.^16^

This study aimed to evaluate the sealing ability of single-cone obturation method with MTA and CEM cement using a bacterial leakage apparatus.

## Materials and Methods

Sixty-four human single-canal canine teeth were obtained from the Department of Oral and Maxillofacial Surgery, Tabriz Faculty of Dentistry; the teeth had been extracted for orthodontic or periodontal reasons. The teeth were examined under a stereomicroscope (Olympus SZ, 9-ILL B200-Choida KU, Japan) and by radiography. The roots with cracks, caries, external or internal resorption and canal calcification were excluded from this study. The teeth were cleaned of tissue remnants and calculus and then rinsed and stored in normal saline. To ensure that all the specimens were of the same length, they were resected at 12-14 mm from the apex using a water-cooled diamond bur (D&Z, Darmstadt, Germany) and then stored in normal saline. All the procedures were performed by a single operator. The canals were accessed and the working length (WL) was determined by inserting a #15 K-file (Mani, Nakanishi Inc., Tokyo, Japan) into the canal until it was just visible at the apical foramen; then 1 mm was subtracted from the measurement. The root canals were prepared using ProTaper rotary Ni–Ti instruments (Dentsply Maillefer, Ballaigues, Switzerland) on an electrical endodontic handpiece (TCM Endo III, Sybron Endo, USA) at 250 rpm. Preparation was carried out according to manufacturer’s recommendations using the crown-down technique. Briefly, the S1 file was used to clean and shape the coronal part of the canal. Subsequently, the SX file was used to increase the taper of the coronal region and S1, S2, F1, F2 and F3 were used sequentially to the full working length. A 15% EDTA gel preparation (Glyde; Dentsply Maillefer) was used as a chelating agent, being introduced into the canal on the tip of each successive instrument. A new set of instruments was used for each group of teeth. None of the instruments fractured during the preparation of the specimens. The canals were irrigated between instruments with 5 mL of freshly prepared solution of 2.5% sodium hypochlorite (NaOCl). Irrigation was performed using 5-mL disposable plastic syringes with 27-gauge needle tips placed passively into the canal, up to 3 mm from the apical foramen without binding. Following instrumentation, the smear layer was removed with 15% EDTA, followed by 2.5% NaOCl. The canal was then dried with sterile paper points. The teeth were randomly divided into 5 groups, consisting of three experimental groups (n=16) and two negative and positive control groups (n=8).

**Figure 1.**
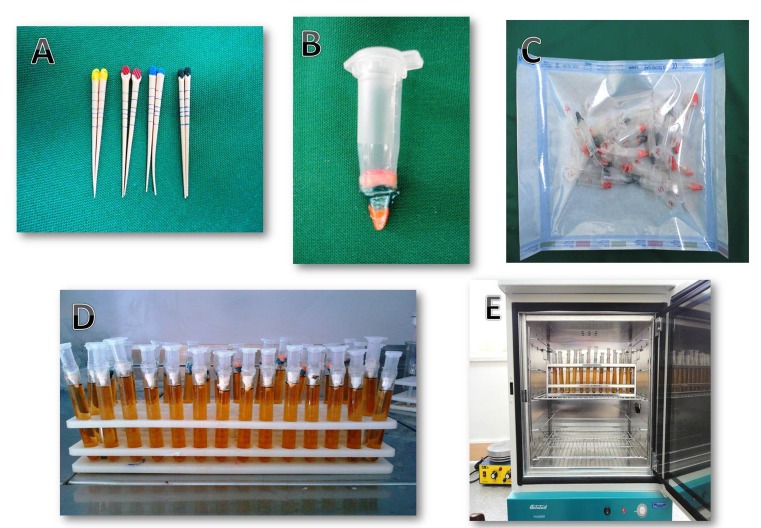
A. Gutta-percha points with greater taper. B. Tooth mounted into the microtube. C. Sterilized and packed samples using ethylene oxide method. D. The apparatus used for bacterial leakage test (the upper chamber for mi-crobial suspension and the lower one for BHI medium). E. Incubation of samples.

In group 1 (AH26/LC), root canal obturation was carried out by gutta-percha (#30, 0.02 taper) and AH26 (Dentsply, DE Trey, Konstanz, Germany) sealer and cold lateral condensation technique. The sealer was applied to canal walls using a lentulo spiral. The master cone was coated with sealer and placed within the canal up to the WL. Then the lateral #20 cones were condensed laterally until the finger spreader #B (Dentsply, Maillefer) could not penetrate further than the coronal third of the canals. In group 2 (MTA/SGP), single Protaper gutta-percha cone (Dentsply Mailleferd) (Figure 1A), matching the size of the F3 file used for final apical preparation, was adjusted in the root canal. Mineral trioxide aggregate (Angelus, Londrina, Brazil) was mixed in low viscosity, like root canal sealers, and applied to canal walls by a lentulo spiral. Gutta-percha was coated with MTA and placed in the canal space up to the WL. In group 3 (CEM/SGP), the same procedure was carried out as that in group 2, except that CEM cement was used as sealer instead of MTA. In group 2 and 3 specimens, no secondary gutta-percha cones or lateral condensation was applied. Excess gutta-percha was removed with a heat carrier and the remaining gutta-percha was vertically compacted at the canal orifice. The access cavities were sealed with Cavit G. In the positive control samples, the teeth were obturated with #30 master gutta-percha without any sealer in order to have maximum bacterial penetration. In the negative control group, the teeth were obturated similar to the group 1 protocol and then immersed in molten wax. The teeth were stored at 37±1°C and 100% relative humidity for 7 days. In all the specimens, except for the negative controls, external root surfaces were covered by two layers of nail varnish except for the apical and coronal portions. In the negative controls, all parts of the specimens were covered by two layers of nail varnish.

###  Bacterial Leakage 

An apparatus, specially developed for the present study, was used. It was based on the straight fitting of 2 tubes: a 2-mL centrifugation microtube and a laboratory glass. The prepared and obturated teeth were fixed to the polyethylene (Figure 1B) microtubes and their interface was sealed by nail varnish and parafilm in order to make them impermeable, but the apical areas of the teeth were left free of varnish. In order to avoid contamination, the apparatus was sterilized using ethylene oxide gas (Figure 1C) before being attached to the glass; the mounting process was performed under a microbial hood using sterile gloves. Then the microtube was inserted into the glass, thus creating two separate chambers (Figure 1D). In the lower one, 2 mm of the root apex remained immersed in the culture medium. In the upper chamber the bacterial suspension was applied. The interface of the microtube and glass was sealed by previously sterilized parafilm, providing lateral impermeability. The apparatus was developed to have a single pathway between the upper chamber (microbial reservoir) and the lower chamber (culture medium), which allowed access to the root canal. This model would permit an assessment of any microbial microleakage that might occur through the root canal sealers.

###  Microbial Preparation 

For the present study, *Enterococcus faecalis* (ATCC 29212) was grown on chocolate agar (Brain Heart Infusion Agar, Oxoid, Basingstoke, UK) for 24 hours at 37°C in CO_2_. Then, *E.faecalis* was inoculated into the tubes containing 5 mL of sterile BHI (Brain Heart Infusion Agar, Oxoid, Basingstoke, UK) suspension and adjusted to a turbidity of 1.5×10^8^ colony-forming units (CFU/mL).

A total of 1 mL of microbial suspensions was placed in the upper chamber of the prepared apparatus. Before bacterial intubation into the upper chamber, the apparatus was incubated for 24 hours at 37°C in order to evaluate sample contamination during the mounting process. If turbidity was observed in the BHI medium, it was considered as contaminated and the previous procedure was repeated. Then, all the glasses were incubated (Figure 1E) at 37°C for 60 days. After 24 hours of incubation, positive and negative controls were checked to ensure the reliability of the test. Every 3 days, 1 mL of the suspension (BHI containing *E.faecalis*) was aspirated from the chamber and replaced by 1 mL of fresh BHI inoculated with *E.faecalis*. The specimens were observed every day for turbidity of the broth in the lower chamber, indicating bacterial growth resulting from penetration of the bacteria past the root canal. To confirm BHI turbidity due to *E. faecalis* growth, the turbid BHI medium was cultured on a chocolate agar medium with a sterile swab; after incubation for 24 hours the colonies were examined and stained with gram staining and studied under a microscope. Then based on hemolysis type, catalase, bile escullin, PYR, optochin disk and 6.5% NaOCl growth tests were performed.^17^ The day of turbidity was recorded. Leakage comparison was carried out between the experimental groups by chi-squared test using SPSS 16 statistical software at a significance level of 0.05.

## Results

**Figure 2.**
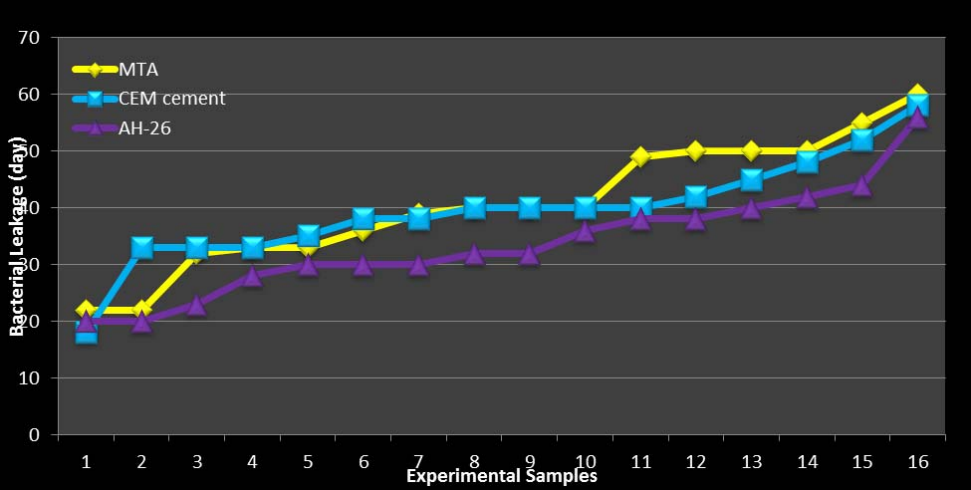
Daily changes of bacterial leakage in the groups studied.

**Figure 3.**
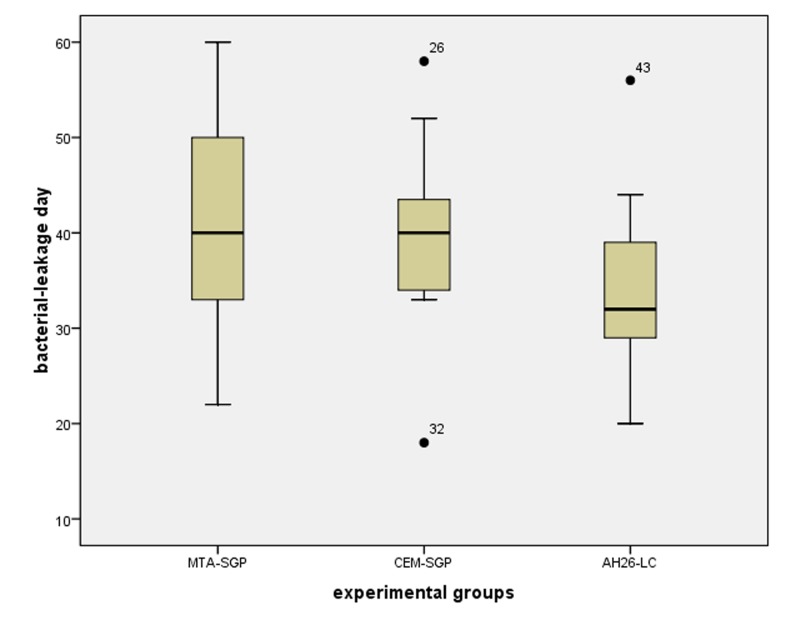
Boxplot of bacterial leakage day of the experimental groups.

In all the positive control specimens, the lower chamber BHI medium exhibited turbidity 24 hours after incubation. During the examination period, none of the negative control specimens exhibited turbidity. *E. faecalis* was isolated from all the turbid specimens by means of enterococcal diagnostic tests (as the colonies were α-hemolytic, opthocin disk, bile escullin, and NaOCl growth tests were carried out). The colonies were resistant to optochin disk, were bile escullin-resistant, and exhibited growth on 6.5% NaOCl medium. Therefore, they were identified as *enterococcus* species. The mean bacterial leakage days in the experimental specimens are presented in Table 1. Kolmogorov-Smirnov analysis revealed that data was normally distributed (P = 0.200). Levene test indicated that the homogeneity of variances matched in the three experimental groups (P = 0.489). Therefore, one-way ANOVA analysis was carried out to compare means between the experimental groups. One-way ANOVA depicted no significant differences in bacterial leakage day between the three experimental groups (P = 0.109, df (2) = 2.328) (Figures 2and 3).

**Table 1:** Statistical data of bacterial leakage of the experimental groups

Experimental groups	N^*^	Mean (Std. Deviation)	95% confidence of interval		P-value		
			Lower	Upper	(One-way ANOVA)	Kolmogorov-Smirnov	Levene Statistic
MTA-SGP	16	40.68 (11.03)	34.80	46.56		0.200	
CEM-SGP	16	39.56 (9.02)	34.75	44.37	P=0.109	0.200	0.489
AH26-LC	16	33.68 (9.39)	28.68	38.69		0.200	

## Discussion

Sealing all the communication pathways between the coronal and apical portions of a root canal system is necessary for the long-term success of root canal therapy. Different materials and techniques have been employed to achieve this goal. Single-cone obturation technique is a less time-consuming technique with better or comparable treatment outcomes in comparison to conventional techniques.^6,18,19^

The thickness of the sealer has been shown to vary depending on the obturation technique. Two concepts are available in single-cone obturation techniques. Some researchers have used this technique with a conventional 0.02% taper master gutta-percha cone and have reported that the single-cone technique requires more sealer and a greater thickness compared to other obturation methods. This increase in sealer volume might lead to an increase in shrinkage, producing more voids and ultimately more leakage.^20-22^ However, others have used this technique by greater tapers of master gutta-percha cones, during which the bulk of the canal system is filled by gutta-percha.^6^Nevertheless, regardless of the technique used in single-cone obturation, it is obvious that leakage takes place through the gaps at sealer-gutta-percha and sealer-canal wall interfaces. Therefore, it seems that there is less microleakage in the second concept because if the bulk of the canal is filled with sealer, the sealer quality and potential to produce voids or gaps within it is important because they can lead to more microleakage; however, it is not true about the second concept as gutta-percha itself is impermeable. This study was performed in relation to the second concept in which rotary ProTaper nickel-titanium files were used to prepare the canals and Protaper single cones for obturation in order to achieve the best fitness of the canal and gutta-percha compared to other preparation techniques.

The most widely used methods in assessing microleakage are the dye penetration technique,^23,24^electrochemical leakage test,^25,26^ and the fluid filtration technique.^27,28^ In several studies limitations of the dye penetration technique have been discussed. Some authors have suggested that the air inside the root canal filling may prevent the penetration of dye.^29,30^ Camps and Pashley reported that dye penetration relies on randomly cutting the root into two pieces without knowing if the section goes through the deepest dye penetration.^31^ The disadvantage of the electrochemical test was pointed out by Amditis et al: corrosion forming on the anodes blocks the diffusion of ions.^25^Bacterial leakage evaluation is a three-dimensional investigation of leakage through the canal system and would better simulate clinical situations. That’s why bacterial leakage was performed to evaluate the sealing efficacy in this study. Different investigators have used various bacterial species for bacterial penetration analysis.^32-35^*Enterococcus faecalis *was used in this study because it is the most virulent bacterial species in the root canal system. Regardless of bacterial species, the important aspect of this test is to confirm that the positive result is attributed to the specific bacterial growth and not to contamination during sample preparation. In this study, the positive samples were confirmed by different isolating tests such as bile escullin, optochin disk, and growth on 6.5% NaOCl medium.

The results of this study showed no differences between single-cone obturation technique using Protaper single cones and MTA or CEM cement as sealer and cold lateral condensation technique by conventional 0.02 taper gutta-percha and AH26 sealer. The factor which is worth considering is that because of good adaptation of gutta-percha to canal walls, only little space remains between gutta-percha and the canal wall, which is filled by the sealer. Therefore, the sealer properties such as viscosity and soaking ability are of utmost importance since it should easily soak gutta-percha and also readily flow in this tiny space to prevent leakage.

In several studies, single-cone technique has been compared to other root canal filling techniques in relation to leakage. Some researchers have compared the sealing ability of single-cone technique and other commonly used ones and have reported no differences between the two techniques.^36-38^ The same findings were revealed in this study, with no significant differences between the techniques evaluated.

## Conclusion

Single-cone obturation with well-fitted gutta-percha and MTA and CEM cement is an alternative for traditional lateral compaction technique.
